# Adjuvant Iodine^**131**^ Lipiodol after Resection of Hepatocellular Carcinoma

**DOI:** 10.1155/2015/746917

**Published:** 2015-12-02

**Authors:** Ruelan V. Furtado, Leo Ha, Stephen Clarke, Charbel Sandroussi

**Affiliations:** ^1^Department of Surgery, Royal Prince Alfred Hospital, Missenden Road, Camperdown, NSW 2050, Australia; ^2^Department of Nuclear Medicine, Repatriation General Hospital, Hospital Road, Concord, NSW 2137, Australia; ^3^Department of Medical Oncology, Royal North Shore Hospital, Reserve Road, St Leonards, NSW 2065, Australia

## Abstract

*Background*. Survival after liver resection for HCC is compromised by a high rate of intrahepatic recurrence. Adjuvant treatment with a single, postoperative dose of intra-arterial I^131^ lipiodol has shown promise, as a means of prolonging disease-free survival (DFS).* Methodology*. DFS and overall survival (OS) after a single dose of postoperative I^131^ lipiodol were compared to liver resection alone, for treatment of hepatocellular carcinoma (HCC). Data were collected retrospectively for patients who had a curative resection for HCC between December 1993 and September 2011. Seventy-two patients were given I^131^ lipiodol after surgery and 70 patients had surgery alone.* Results*. The DFS at 1, 3, and 5 years was 72%, 43%, and 26% in the surgery group and 70%, 39%, and 29% in the adjuvant I^131^ lipiodol group (*p* = 0.75). The 1-, 3-, and 5-year OS was 83%, 64%, and 52% in the surgery group and 96%, 72%, and 61% in the adjuvant I^131^ lipiodol group (*p* = 0.16).* Conclusion*. This retrospective study has found no significant benefit to survival, after adjuvant treatment with I^131^ lipiodol.

## 1. Introduction

Hepatocellular carcinoma (HCC) is the third-leading cause of cancer mortality worldwide, with an increasing incidence in the West [1–3]. While liver transplantation offers the best hope of cure, it is constrained by the availability of donor organs worldwide [4]. Hence liver resection remains the mainstay of treatment. Survival is limited however by, on average, a 60% long-term rate of intrahepatic recurrence [5].

Lipiodol is an ester of fatty acids derived from poppy seed oil that has been used to diagnose and treat HCC. This compound was initially used as a radiological contrast medium and was found to have higher uptake in HCC, relative to normal liver tissue [6]. It contains an iodine^127^ moiety, which can be exchanged for iodine^131^ (I^131^), to create a compound that delivers targeted, internal, beta, and gamma radiation. Early studies showed that injection of I^131^ lipiodol into the hepatic artery at angiography could induce tumor necrosis and significantly prolong survival in inoperable patients [7]. The treatment has been used since the 1990s as palliation for HCC, as it is well-tolerated with few complications or side effects [8, 9]. Its use as an adjuvant treatment is less established.

Long-term data from 2 RCTs have not provided evidence for the use of adjuvant I^131^ lipiodol after curative resection for HCC. On the other hand, multiple retrospective studies have supported its use. A meta-analysis of all published studies was recently published by the authors of this paper. This suggested a significant survival benefit to adjuvant treatment with I^131^ lipiodol, 5 years after surgery [10]. The current study was done to add to the database of patients, who have had this treatment.

## 2. Aims

A retrospective study was undertaken of all patients undergoing a curative liver resection for HCC at Royal Prince Alfred Hospital in Sydney. Patients who received surgery and adjuvant I^131^ lipiodol were compared to those who had a resection only. The outcome factors were disease-free survival (DFS) and overall survival (OS).

## 3. Patients and Methods

### 3.1. Allocation and Perioperative Management

All patients with HCC confined to the liver were reviewed at a multidisciplinary meeting. Allocation to ablation, liver resection, or transplant was by Milan criteria [11] in the years 1994–2003 and by University of California, San Francisco criteria [12] from 2004 onwards. The standard preoperative workup included multiphase, computed tomography (CT), or contrast-enhanced magnetic resonance imaging (MRI). Liver biopsy was not used in any of the patients in this study. Major vascular invasion seen on imaging or at laparotomy was not a contraindication to resection. Patients were excluded if there were positive microscopic margins and if they died within 30 days.

Where there was concern about liver function, angiography with hepatic venous pressure gradients (HVPG) was used. Eligibility for a major liver resection was limited to disease confined to the liver, preserved liver function (Child-Pugh grade A [13]), and a HVPG less than 12 mmHg [14, 15]. Those with cirrhosis and poorer liver function were limited to a nonanatomical resection or nonoperative treatment if the former was not possible.

Laparotomy with intraoperative ultrasound was standard in almost all cases. At this stage, resection was deferred if there were unsuspected metastases or extensive disease in the presence of cirrhosis. Total vascular isolation or inflow occlusion was used selectively. Knife resection, ultrasonic aspirator, or hydrojet was used for liver parenchymal dissection. A major liver resection was defined as removal of 3 or more Couinaud segments.

### 3.2. Adjuvant I^131^ Lipiodol

Allocation to treatment with adjuvant I^131^ lipiodol was by surgeon or oncologist preference in consultation with patients. I^131^ lipiodol was obtained commercially (Ansto Health, Lucas Heights, Australia). An interventional radiologist gained access to the hepatic artery and the medication was administered by a nuclear medicine physician. A test dose of Technetium^99^ macroaggregated albumin was given immediately prior to the treatment dose of lipiodol, to assess for arterial anomalies or pulmonary shunting.

Patients with significant flow to the lungs had their dose adjusted, to minimize the risk of radiation pneumonitis. Patients were excluded, in whom there was a significant arterial anomaly predisposing to extrahepatic I^131^ deposition. Other exclusion criteria were severe allergy to iodine, severe renal disease, pregnancy, and breastfeeding.

Treatment with the full dose was based on the remnant liver volume in early years and was changed to 2.0 GBq per patient after 2010. Patients spent 1 night in hospital and were discharged home for 6 days of isolation. A gamma camera image was taken prior to discharge to assess for abnormal deposition of I^131^. Lugol's iodine was given 5 days prior to and 1 week following treatment, to protect the thyroid.

### 3.3. Follow-Up

Screening for recurrence was done with CT at 6 months after surgery by the surgeon and then patients were either referred to the liver outpatient clinic or their general practitioner. Biannual imaging with CT was standard follow-up.

Recurrence was defined as a hypervascular lesion on CT with early washout that was confirmed by a second imaging modality. These included MRI, ultrasound scan, or angiography [16, 17]. If two imaging modalities were not available, a single study suggesting recurrence associated with a serum alpha-fetoprotein (AFP) greater than 6.0 IU/mL was diagnostic. Suspicious extrahepatic recurrences were biopsied, for histological confirmation. The date of recurrence was taken when any of the above criteria were first met. Patients who died prior to imaging follow-up were censored at the time of surgery.

### 3.4. Data Collection

Retrospective data were collected for consecutive patients who had surgery between the years 1993 and 2011. Hospital, surgeon, general practitioner, and population records were searched. Exclusion criteria were macroscopically positive margins (*n* = 6), microscopically positive margins (*n* = 23), missing data (*n* = 17), and death within 30 days of surgery (*n* = 6) or if the initial liver resection was performed elsewhere (*n* = 9). In addition, four patients were excluded because of atypical pathology showing fibrolamellar HCC (*n* = 1), cholangio-HCC (*n* = 2), or HCC with a synchronous liver adenocarcinoma (*n* = 1).

Data were collected regarding patient age, sex, ethnicity, and the etiology of underlying chronic liver disease. The presence of major vascular invasion was noted on preoperative imaging or in the operative notes. Significant pathological details included resection margin, focality, microvascular invasion, nodal status, grade, preoperative AFP, and the presence and degree (Child-Pugh class [13]) of cirrhosis. Tumor size was taken as the maximum diameter of the largest tumor nodule, in the resected specimen. The AJCC 2010 stage [18] was calculated for each patient based on supplied pathological data. The date and variety of palliative treatments were recorded, as well as the calculated activity of I^131^ for each patient.

### 3.5. Statistical Analysis

Statistics were carried out using SPSS version 23 (IBM SPSS, New York, USA). Fisher's exact test was used to compare categorical data. Survival was compared using Kaplan Meier analysis using the log rank test. Cox proportional hazards were used. Clinically important variables and those with *p* value less than or equal to 0.1 on univariate analysis were included in a multivariate analysis. Median follow-up was calculated by reverse Kaplan-Meier analysis [19]. Significance was accepted at *p* < 0.05.

## 4. Results

There were 52 patients, who had a surgical resection only, and 58 patients had a resection and adjuvant, I^131^ lipiodol. The baseline demographics, tumor characteristics, and chronic liver disease status of these patients are shown in Table 1. The median activity of I^131^ lipiodol administered was 1.8 GBq (range 0.9–3.6) and treatment was administered at a median of 86 days after surgery. Age, sex ratio, tumor size, multifocality, microvascular invasion, differentiation, margin status, etiology, and stage of chronic liver disease were similar, in both groups. Significantly more patients of Asian ethnicity received adjuvant treatment with lipiodol (28 versus 40, *p* = 0.046).

Two adverse events occurred, as a consequence of allocation to adjuvant treatment. An unrecognised, arterial anomaly leads to deposition of I^131^ in the gastric antrum. A second patient was observed to have asymptomatic uptake in the muscles of the lower limb. Both patients were observed as inpatients, and the first was given oral proton pump inhibitors. No adverse outcome occurred in either case.

**Figure 1:**
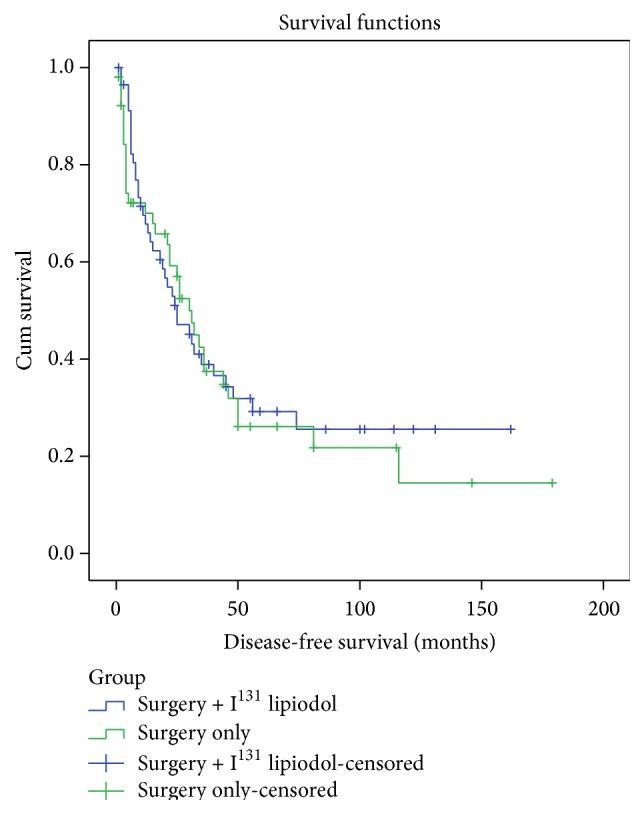
Disease-free survival after resection of HCC, surgery only versus surgery with adjuvant I^131^ lipiodol.

The median follow-up period was 66 months (95% CI, 36–96 months). During this time, there were 35 (67%) recurrences in the surgery only group and 38 (66%) recurrences in the adjuvant I^131^ lipiodol group (HR 0.93, 95% CI 0.59–1.5, *p* = 0.75). The number of intrahepatic recurrences was 29 (56%) in the surgery only group and 33 (57%) in the adjuvant group. The median DFS was 30 (95% CI, 22–38) months in the surgery group and 25 (95% CI, 14–36) months in the surgery and I^131^ lipiodol group (*p* = 0.74). The 1-, 3-, and 5-year DFS were 72% (95% CI, 60–84%), 43% (95% CI, 29–57%), and 26% (95% CI, 12–40%) in the surgery group and 70% (95% CI, 58–82%), 39% (95% CI, 25–53%), and 29% (95% CI, 15–43%) in the adjuvant lipiodol group (Figure 1).

**Figure 2:**
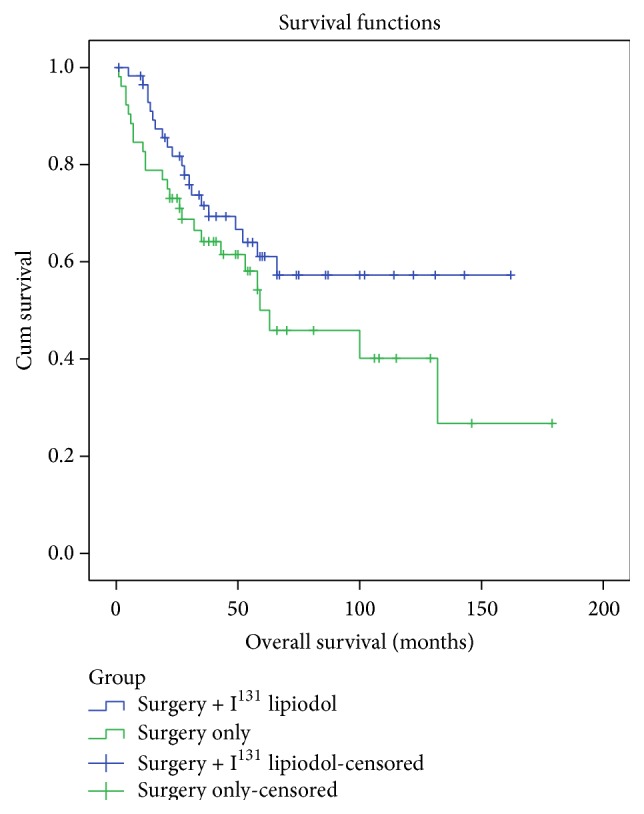
Overall survival after resection of HCC, surgery only versus surgery with adjuvant I^131^ lipiodol.

Twenty-five (48%) patients died in the surgery only group and 20 (34%) died in the adjuvant lipiodol group (HR 0.66, 95% CI, 0.37–1.2, *p* = 0.16, Figure 2). The median overall survival for the surgery only group was 63 (95% CI, 18–107) months and median survival time was not reached in the adjuvant lipiodol group (*p* = 0.16). The 1-, 3-, and 5-year OS were 83% (95% CI, 73–93%), 64% (95% CI, 50–78%), and 52% (95% CI, 36–68%) in the surgery group and 96% (95% CI, 92–100%), 72% (95% CI, 60–84%), and 61% (95% CI, 47–75%) in the adjuvant lipiodol group.  Table 2 shows the treatments given on diagnosis of disease recurrence. Patients in the treatment group had significantly more repeat liver resections (13 versus 4), on diagnosis of intrahepatic recurrence (*p* = 0.034). Conversely, patients in the control group were more likely to have medical treatments (i.e., either sorafenib, sandostatin, temozolomide, or thalidomide) than the treatment group (9 versus 1, *p* = 0.005) on diagnosis of recurrence.

Univariate Cox regression analysis of 10 variables affecting DFS is shown in Table 3. Factors associated with survival included AJCC stage (*p* = 0.001), multifocality (*p* = 0.001), and microvascular invasion (*p* = 0.001, Table 3). Multivariate analysis showed that there was no variable that was an independent predictor for poorer DFS associated with resection (Table 4).

Univariate Cox regression analysis of the same variables affecting OS is shown in Table 3. Factors associated with survival were AJCC stage (*p* = 0.001), differentiation (*p* = 0.001), multifocality (*p* = 0.001), and microvascular invasion (*p* = 0.001). These variables and ethnicity (*p* = 0.091) were analysed in multivariate regression. Multifocality (HR 3.5, 95% CI 1.6–7.6), microvascular invasion (HR 2.6, 95% CI 1.2–4.4), and differentiation (HR 2.2, 95% CI 1.3–3.7) were associated with poorer OS (Table 4).

## 5. Discussion

This study does not provide evidence that adjuvant I^131^ lipiodol alters DFS or OS after resection of HCC. The cohort is the largest sample studied to date, and the median follow-up is just greater than 5 years. The findings suggest that the role of adjuvant treatment may not be as broad as initially suggested by the landmark Hong Kong trial (HKT) [20].

The early results of the HKT provided strong evidence that patients given a single dose of adjuvant I^131^ lipiodol after resection had longer DFS and OS compared to those who only had resection. With a median follow-up of 34 months at initial reporting, the hazard ratio (HR) for DFS was 2.7 (95% CI 1.0–7.1) and OS 3.1 (1.0–9.9). Thus there was a significant survival benefit, although the wide confidence intervals suggested the strength of this effect was uncertain.

A follow-up paper at 66 months showed this survival benefit disappeared at 8 years after randomization. There was evidence, however, that I^131^ lipiodol significantly delayed the onset of intrahepatic recurrence from 7 to 19 months. The HKT stopped accruing patients early due to the strong survival benefit seen on interim analysis. Ultimately it was underpowered; hence the failure to detect a long-term benefit may have been due to type 2 error [21].

A recent, large, multicentre RCT from Singapore (designated AHCC03) also found an insignificant benefit to survival, after a median follow-up of 80 months. Although it remains the largest prospective study to date (*n* = 103), it too failed to accrue enough subjects and was powered at 50%. The authors concluded that treatment may prolong survival, but the trial itself could not demonstrate this [22]. Hence the data from RCTs do not support a survival benefit with adjuvant I^131^ lipiodol.

On the other hand, multiple retrospective studies of adjuvant lipiodol have demonstrated significantly improved DFS in patients treated with adjuvant I^131^ lipiodol [2, 23, 24]. The populations studied in these trials were different from the RCTs in that more advanced tumours, higher grade cirrhosis, and macroscopically positive margins were included in the analyses. It is unclear why these populations, which objectively have a worse prognosis, have better DFS after treatment with I^131^ lipiodol.

The results of the current paper are different from the previously published retrospective studies in that no significant benefit to survival was shown. The current study had patients with a relatively high rate of microvascular invasion (34%), similar to the AHCC03 trial (28%). This is significantly greater than the HKT (4.6%), and this may be the factor that renders adjuvant treatment with I^131^ lipiodol less effective. It is conceivable that a targeted, arterially delivered therapy may be less effective, if tumour cells have already spread into the bloodstream.

There were some significant confounders in this study, owing to its retrospective nature. Significantly more patients in the treatment arm had repeat liver resections for intrahepatic recurrence. On the other hand, patients in the control group were more likely to have adjuvant medical therapy. This likely represents selection bias. At the study institution patients referred for adjuvant lipiodol treatment are often followed up by surgeons, while other patients are seen by oncologists. Significantly more patients of Asian ethnicity were referred for treatment with I^131^ lipiodol as well; however ethnicity was not found to be a significant covariate on either univariate or multivariate regression. Furthermore, the aetiology of chronic liver disease was similar in both groups, despite the racial difference. It is unclear why more Asian patients would be referred for treatment with lipiodol and it may represent bias in referrer or patient preference.

## 6. Conclusion

Results from this study do not support the routine use of adjuvant, radiolabelled lipiodol following excision of HCC to prolong survival. A high rate of microvascular invasion may suggest that treatment with adjuvant lipiodol is less likely to be effective. Adequately powered, randomised trials could address this question in the future.

## Figures and Tables

**Table 1:** Patient characteristics.

	Surgery only	Surgery + I^131^-lipiodol	Significance (*p* value)
Number	52	58	
Age, mean and SD	58 (14)	55 (12)	0.23
Sex, *n* (M/F)	36/16	44/14	0.29
AJCC (2010) stage, *n*			
1	23	32	0.34
2	14	17	0.83
3a	9	5	0.25
3b	1	2	1.0
3c	5	2	0.25
Tumor size (mm), median and range	46.5 (15–150)	35.0 (15–110)	0.55
Resection margin (mm), median and range	5.5 (0.050–43)	5 (0.10–45)	0.81
Multifocality, *n*	20	15	0.22
Microvascular invasion, *n*	18	19	0.50
Major vascular invasion, *n*	2	2	
Differentiation, *n*			
Well	10	11	1.0
Moderate	32	33	0.70
Poor	9	14	0.35
Not stated/unknown	1	0	0.47
Race, *n*			
Asian	28	40	0.042
Caucasian	20	14	0.15
Other	4	4	1.0
Chronic liver disease, *n*			
Hepatitis B	27	34	0.57
Hepatitis C	11	16	0.51
Other	14	8	0.10
Child-Pugh stage, *n*			
A	51	58	0.47
B	1	0	
Cirrhosis, *n*	26	31	0.43
Serum AFP, median and range	10.75 (0–13241)	28 (1–7281)	0.39

**Table 2:** Treatment details.

	Surgery only	Surgery + I^131^-lipiodol	Significance (*p* value)
Dose of I^131^ lipiodol (GBq)	—	1.8	
Liver resection, *n*			0.56
Major	22	21	
Minor	30	37	
Deaths, *n*	25	20	0.16
Recurrences, *n*	34	38	
Intrahepatic	29	33	1.0
Lung	3	3	1.0
Bone	2	1	0.60
Other	0	1	1.0
Treatment of recurrence, *n*			
Transarterial chemoembolization (TACE)	12	12	0.81
Repeat liver resection	4	13	0.038
Salvage liver transplant	4	4	1.0
Ablation	9	9	0.79
DC bead TACE	2	3	1.0
Percutaneous ethanol	6	3	0.29
Palliative I^131^ lipiodol	1	2	1.0
External beam radiotherapy	0	3	0.24
Sorafenib	3	1	0.005
Systemic chemotherapy	3	0	
Sandostatin	2	0	
Temozolomide	1	0	

**Table 3:** Results of univariate Cox regression for 12 patient variables.

Patient characteristic	*p* value, OS	*p* value, DFS
AJCC (2010) stage	0.001	0.001
Ethnicity	0.091	0.46
Differentiation	0.001	0.063
Multifocality	0.001	0.001
Etiology of CLD	0.24	0.27
Sex	0.91	0.70
Age (>50)	0.86	0.44
Tumor size (>20 mm)	0.98	0.31
Microvascular invasion	0.001	0.018
Cirrhosis (yes/no)	0.90	0.65

**Table 4:** Results of multivariate Cox regression for OS and DFS.

Patient characteristic (OS)	Multivariate Cox regression *p* value	Hazard ratio (95% CI)
Multifocality	0.002	3.5 (1.6–7.6)
Microvascular invasion	0.01	2.3 (1.2–4.4)
Differentiation	0.002	2.2 (1.3–3.7)
AJCC stage	0.72	1.1 (0.77–1.5)
Ethnicity	0.44	0.82 (0.5–1.4)

Patient characteristic (DFS)	Multivariate Cox regression *p* value	Hazard ratio (95% CI)

Multifocality	0.17	1.6 (0.82–3.1)
Microvascular invasion	0.49	1.2 (0.71–2.1)
Differentiation	0.17	1.3 (0.89–1.9)
AJCC stage	0.07	1.3 (0.97–1.7)

## References

[1] Altekruse S. F., McGlynn K. A., Reichman M. E. (2009). Hepatocellular carcinoma incidence, mortality, and survival trends in the United States from 1975 to 2005. *Journal of Clinical Oncology*.

[2] Boucher E., Bouguen G., Garin E., Guillygomarch A., Boudjema K., Raoul J.-L. (2008). Adjuvant intraarterial injection of ^131^I-labeled lipiodol after resection of hepatocellular carcinoma: progress report of a case-control study with a 5-year minimal follow-up. *Journal of Nuclear Medicine*.

[3] Breedis C., Young G. (1954). The blood supply of neoplasms in the liver. *The American Journal of Pathology*.

[4] Dhir M., Lyden E. R., Smith L. M., Are C. (2012). Comparison of outcomes of transplantation and resection in patients with early hepatocellular carcinoma: a meta-analysis. *HPB*.

[5] Abdalla E., Stuart K., Savarese D. (2013). Overview of treatment approaches for hepatocellular carcinoma. *UpToDate*.

[6] Park C. H., Suh J. H., Yoo H. S., Lee J. T., Kim D. I. (1986). Evaluation of intrahepatic I-131 ethiodol on a patient with hepatocellular carcinoma. Therapeutic feasibility study. *Clinical Nuclear Medicine*.

[7] Yoo H. S., Lee J. T., Kim K. W. (1991). Nodular hepatocellular carcinoma. Treatment with subsegmental intraarterial injection of iodine 131-labeled iodized oil. *Cancer*.

[8] Kobayashi H., Hidaka H., Kajiya Y. (1986). Treatment of hepatocellular carcinoma by transarterial injection of anticancer agents in iodized oil suspension or of radioactive iodized oil solution. *Acta Radiologica: Diagnosis*.

[9] Raoul J.-L., Guyader D., Bretagne J.-F. (1994). Randomized controlled trial for hepatocellular carcinoma with portal vein thrombosis: intra-arterial iodine-131-iodized oil versus medical support. *Journal of Nuclear Medicine*.

[10] Furtado R., Crawford M., Sandroussi C. (2014). Systematic review and meta-analysis of adjuvant I131 lipiodol after excision of hepatocellular carcinoma. *Annals of Surgical Oncology*.

[11] Mazzaferro V., Rondinara G. F., Rossi G. (1994). Milan multicenter experience in liver transplantation for hepatocellular carcinoma. *Transplantation Proceedings*.

[12] Yao F. Y., Ferrell L., Bass N. M., Bacchetti P., Ascher N. L., Roberts J. P. (2002). Liver transplantation for hepatocellular carcinoma: comparison of the proposed UCSF criteria with the Milan criteria and the Pittsburgh modified TNM criteria. *Liver Transplantation*.

[13] Pugh R. N. H., Murray Lyon I. M., Dawson J. L. (1973). Transection of the oesophagus for bleeding oesophageal varices. *The British Journal of Surgery*.

[14] Bruix J., Castells A., Bosch J. (1996). Surgical resection of hepatocellular carcinoma in cirrhotic patients: prognostic value of preoperative portal pressure. *Gastroenterology*.

[15] Boleslawski E., Petrovai G., Truant S. (2012). Hepatic venous pressure gradient in the assessment of portal hypertension before liver resection in patients with cirrhosis. *The British Journal of Surgery*.

[16] Yamamoto J., Kosuge T., Takayama T. (1996). Recurrence of hepatocellular carcinoma after surgery. *The British Journal of Surgery*.

[17] Sumie S., Kuromatsu R., Okuda K. (2008). Microvascular invasion in patients with hepatocellular carcinoma and its predictable clinicopathological factors. *Annals of Surgical Oncology*.

[18] Edge S. B., Compton C. C. (2010). The American Joint Committee on Cancer: the 7th edition of the AJCC cancer staging manual and the future of TNM. *Annals of Surgical Oncology*.

[19] Schemper M., Smith T. L. (1996). A note on quantifying follow-up in studies of failure time. *Controlled Clinical Trials*.

[20] Lau W. Y., Leung T. W. T., Ho S. K. W. (1999). Adjuvant intra-arterial iodine-131-labelled lipiodol for resectable hepatocellular carcinoma: a prospective randomised trial. *The Lancet*.

[21] Lau W. Y., Lai E. C. H., Leung T. W. T., Yu S. C. H. (2008). Adjuvant intra-arterial iodine-131-labeled lipiodol for resectable hepatocellular carcinoma: a prospective randomized trial—update on 5-year and 10-year survival. *Annals of Surgery*.

[22] Chung A. Y. F., Ooi L. L. P. J., Machin D. (2013). Adjuvant hepatic intra-arterial iodine-131-lipiodol following curative resection of hepatocellular carcinoma: a prospective randomized trial. *World Journal of Surgery*.

[23] Tabone M., Vigano L., Ferrero A., Pellerito R., Carbonatto P., Capussotti L. (2007). Prevention of intrahepatic recurrence by adjuvant ^131^iodine-labeled lipiodol after resection for hepatocellular carcinoma in HCV-related cirrhosis. *European Journal of Surgical Oncology*.

[24] Chua T. C., Chu F., Butler S. P. (2010). Intra-arterial iodine-131-lipiodol for unresectable hepatocellular carcinoma. *Cancer*.

